# Pt(II)-Thiocarbohydrazone Complex as Cytotoxic Agent and Apoptosis Inducer in Caov-3 and HT-29 Cells through the P53 and Caspase-8 Pathways

**DOI:** 10.3390/ph14060509

**Published:** 2021-05-26

**Authors:** Abeer A. Ibrahim, Mohanad M. Kareem, Taghreed H. Al-Noor, Tahani Al-Muhimeed, Abeer A. AlObaid, Salim Albukhaty, Ghassan M. Sulaiman, Majid Jabir, Zainab J. Taqi, Usama I. Sahib

**Affiliations:** 1 Department of Medical Laboratories Science, Technical College of Health, Sulaimani Polytechnic University, Sulaymaniyah 46001, Iraq; abeer.ibrahim@spu.edu.iq; 2 Department of Chemistry, College of Science, University of Babylon, Babil-Hilla 51002, Iraq; sci.mohanad.mousa@uobabylon.edu.iq; 3 Department of Chemistry, College of Education for Pure Sciences/Ibn al-Haitham, University of Baghdad, Baghdad 10053, Iraq; drtaghreed2@gmail.com; 4 Department of Chemistry, College of Science, King Saud University, P.O. Box 22452, Riyadh 11495, Saudi Arabia; talmuhimeed@ksu.edu.sa (T.A.-M.); aaalobaid@ksu.edu.sa (A.A.A.); 5 Department of Chemistry, College of Science, University of Misan, Amarah 62001, Iraq; albukhaty.salim@uomisan.edu.iq; 6 Department of Applied Sciences, University of Technology, Baghdad 10066, Iraq; 100293@uotechnology.edu.iq (Z.J.T.); usamaimad3@gmail.com (U.I.S.)

**Keywords:** Pt(II) complex, anticancer activity, apoptosis, DNA fragmentation, p53, caspase-8

## Abstract

In this study, a platinum(II) complex ([Pt(H_2_L)(PPh_3_)] complex) containing a thiocarbohydrazone as the ligand was tested as an anti-proliferative agent against ovarian adenocarcinoma (Caov-3) and human colorectal adenocarcinoma (HT-29) through MTT assays. Apoptotic markers were tested by the AO/PI double staining assay and DNA fragmentation test. Flow cytometry was conducted to measure cell cycle distribution, while the p53 and caspase-8 pathways were tested via immunofluorescence assay. Results demonstrated that the cytotoxic effect of the Pt(II)-thiocarbohydrazone complexes against Caov-3 and HT-29 cells was highly significant, and this effect triggered the activation of the p53 and caspase-8 pathways. Besides, apoptosis stimulated by the Pt(II)-thiocarbohydrazone complex was associated with cell cycle arrest at the G0/G1 phase. These findings suggest that the target complex inhibited the proliferation of Caov-3 and HT-29 cells, resulting in the arrest of the cell cycle and induction of apoptosis via the stimulation of the p53 and caspase-8 pathways. The present data suggests that the Pt(II)-thiocarbohydrazone complex could also be a promising chemotherapeutic agent for other types of cancer cells.

## 1. Introduction

Cancer is a critical disease of interest to scientists due to its long history of being among the leading causes of death [1]. There is no single disorder representing cancer; rather, it is a collection of disorders marked by the uncontrollable outgrowth of cells. Tumors are a serious risk of lethal disease with no geographic location or organ limits; they induce an annual worldwide mortality exceeding 12.7 million individuals. Mutated genes that regulate growth and are involved in DNA repair, cell division, and death typically causes tumor diseases [2].

Cancer can occur accidentally when a portion of the genetic code is miscopied, whereas DNA damage by chemical, viral, or radiation exposure may lead to cancer induced by the environment. Previous studies on the development of anticancer strategies include various instances related to the interactions of metal ions or metal-containing compounds with biological targets. Selecting the ligand in a metal complex is as essential as selecting the metal due to its effectiveness on biological systems [3,4,5,6]. In pharmacology, heterocyclic compounds play a significant role in the composition of the drug formula. 

Purine, thiosemicarbazone, imidazole, and benzohydroxamic ligands containing different donor atoms that can chelate metal ions can exert different anti-cancer functions. In vitro and in vivo investigations of several anticancer drugs containing metal ions showed more potent antineoplastic actions than that of cisplatin [7,8,9,10]. Anticancer drugs based on platinum (Pt) still constitute an essential part of vast regimens of chemotherapy [4]. Although they cause toxicity as a part of their side effects, they also lead to drug resistance [11,12,13]. Meanwhile, the mechanism of action of Pt against cancer cell lines remains unclear. The three foremost steps related to antitumor action are as follows: cell uptake of drugs; the interplay of the drug with DNA, including intervention with the activities of transcription and/or replication; and the platinum-DNA lesion that evokes cell death, which depends on the activation of the signal transduction pathways [14]. 

The cytotoxicity of platinum-based drugs induced by the activated platinum species binding to intracellular targets is predominantly dependent on DNA. Following the recognition of the DNA adducts by the cellular systems, three concurrent potential pathways are involved. In the first pathway, DNA can be repaired. In the second, the damaged DNA fragment can be bypassed. In the third, apoptosis can take place. Inhibition of transcription assumes the common way to evoke cell apoptosis after treatment with Pt compounds [15]. Based on the potency of platinum complexes as anticancer agents to obtain new more effective and less toxic drug candidates than cisplatin, this work explores the activity of a Pt(II)-thiocarbohydrazone complex as a potential anticancer agent. The attractiveness of thiocarbohydrazones for developing new metal-based drugs relies on their structural diversity providing interesting coordination chemistry and promising biological implications [16,17]. 

The Caov-3 cell line is an epithelial morphology main line of ovarian cancer cells. They are susceptible to adriamycin, cisplatin, and vinblastine. Improvements in ovarian cancer outcomes are expected, particularly for ovarian clear cell carcinomas (OCCA), from a clear understanding of molecular pathology that may drive early detection approaches and effective treatment [18]. Thus, the present study was performed to evaluate the anti-proliferative and pro-apoptotic effects of the Pt(II)-thiocarbohydrazone complex on Caov-3 and HT-29 cell lines. Our results explained the possible mechanisms involved in the effect of this complex on the used cell lines, namely, mitochondrial damage and the p53 and caspase-8 pathways.

## 2. Results and Discussion

### 2.1. Pt(II) Complex as Anti-Proliferative Agent against Caov-3 and HT-29 Cells

**Figure 1:**
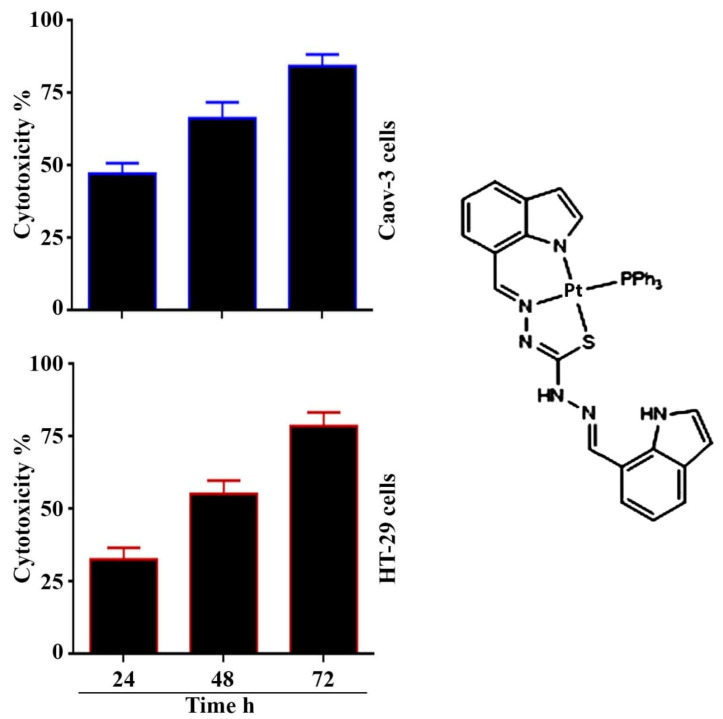
(left lane) Cytotoxicity of [Pt(H_2_L)(PPh_3_)] against Caov-3 and HT-29 cells at different time (24, 48 and 72 h). IC_50_ for Caov-3 cells were 3.34, 2.41, 1.74 μg mL^−1^, respectively. While, for HT-29 cells were 10.32, 4.25, 2.5 μg mL^−1^, respectively. (right lane) Structure of Pt(II) complex.

In this study, we measured the cytotoxic effect of the Pt(II) complex at different times as shown in Figure 1. The IC_50_ values of the Pt(II) complex following 24 h exposure of the cancer cell line was 3.34 μg mL^−1^ for Caov-3 and 10.32 μg mL^−1^ for HT-29. Whereas, the IC_50_ values for 48 h exposure to the complex were 2.41 μg mL^−1^ for Caov-3 and 4.25 μg mL^−1^ for HT-29 cancer cell lines. The IC_50_ values for 72 h exposure were 1.74 μg mL^−1^ for Caov-3 and 2.5 μg mL^−1^ for HT-29 cancer cell lines. These results indicated that prolonged treatment significantly reduced the IC_50_. These results agree with the previous study of Jain et al. [19].

The activity of the here-presented Pt complex was compared with that of cisplatin (a clinically-used anticancer drug) in analogy to other works on platinum complexes [20,21]; the IC_50_ values of [Pt(H_2_L) (PPh_3_)], presented in Figure 1, resulted lower demonstrating greater cytotoxic effects than cisplatin towards the same cancer cell lines. While other study of Paschke et al. verified that the CholCOO(CH_2_ )n CH(CH_2_ NH_2_ ) Pt_2_ CBDC (carbo-ChAPt) as well as the CholCOO(CH_2_)n CH(CH_2_ NH_2_ ) PtCl_2_ (cis- ChAPt) showed similar cytotoxicity against testicular cancer cells as their partner compounds carboplatin and cisplatin [11]. 

### 2.2. Pt(II) Complex Induces Apoptosis in Caov-3 and HT-29 Cells

**Figure 2:**
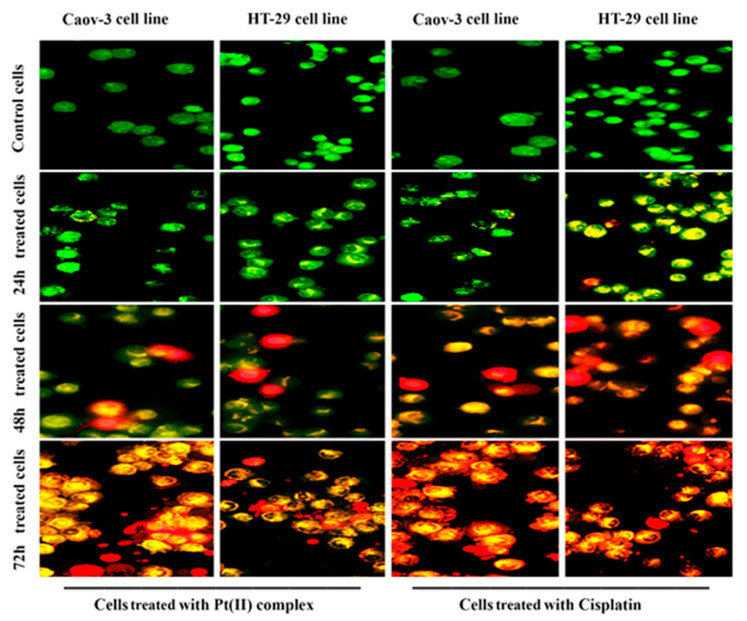
Fluorescence microscopy images of cell lines stained with AO and PI. Cells were treated with IC_50_ concentrations of [Pt(H_2_L)(PPh_3_)] complex at different times.

The (AO/PI) dual stain is a fluorescent combination stain that enables to detection of morphological modification in the nucleus by producing distinctive fluorescent colors. Apoptotic cells have increased fluorescence stain permeability to the plasma membrane. After treating the cell lines with the IC_50_ concentrations of the tested compound for 24, 48, and 72 h, dual cell staining and fluorescence microscope visualization were performed to recognize changes in nuclear morphology [21]. The treatment of cells with IC_50_ concentrations of the [Pt(H_2_L)(PPh_3_)] complex led to disruption of the membrane and vacuoles of the lysosomes compared with untreated cells with effects increased with the incubation time (Figure 2). The same results were observed after treated the Caov-3 and HT-29 cells with cisplatin. The results showed the high potential of the platinum complex to cause cell death due to the ability of the molecules to penetrate through the membrane [22,23]. A mixture of AO/PI dyes was used to further examine the potential of the tested compound to induce cancer cell death. In this process, the structure of the nuclei of the cells was observed to be intact, with a stable bright green color. Compared with the membranes of untreated cells, the membranes of cancer cells treated with the test compounds exhibited less integrity apoptotic cells typically display nuclei that are distinguished by their red to green color, while their chromatin condensation differs. The morphological changes in the treated cells indicated that apoptosis rather than necrosis caused the observed cell death. Through the combination of the AO stain into DNA, cells suffering from early cell death or apoptosis were identified, leading to the emission of bright green fluorescence after 24 h. Mild apoptosis was observed during therapy in the form of condensed chromatin and membrane blebbing, which are apoptosis-related time-dependent morphological features. Additionally, the same dose of the Pt(II) complex caused late apoptosis events after 48 and 72 h that were recognized through many changes that included binding between two stains and damaged DNA, leading to the emission of a reddish-orange color. In the present work, we measured the possibility that inducing apoptosis was related to the anti-proliferation events in the tested cancer cell line Caov-3 and HT-29 after treatment with the Pt(II) complex.

### 2.3. Pt(II) Complex Causes DNA Fragmentation in Caov-3 and HT-29 Cells

**Figure 3:**
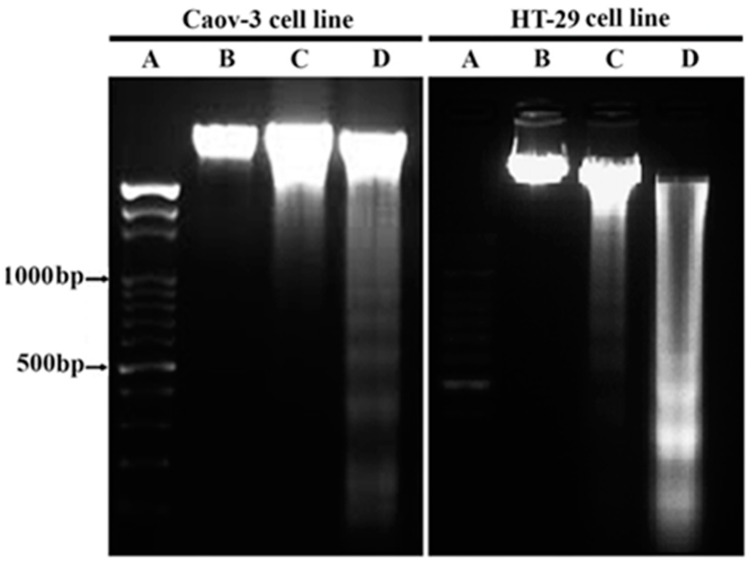
DNA fragmentation assay. Lane A: 1000 bp molecular weight marker, Lane B: untreated cells, Lane C: treated cells for 48 h, Lane D: treated cells for 72 h.

The formation of nucleosome units from the breakdown of DNA is one of the main characteristics of apoptosis. To identify the fragmentation of the nucleic acid inside the cancer cells, the ovarian adenocarcinoma and colorectal adenocarcinoma cell lines (Caov-3 and HT-29, respectively) were incubated with the corresponding IC_50_ values of the Pt- complex for 48 and 72 h. After lysing and extracting the nucleic acid of the cell lines, gel electrophoresis was used to identify the changes in the nucleic acid. As shown in Figure 3, a comparison of nucleic acid fragmentation at 72 h for both cell lines showed a ladder range from 500 bp to 1000 bp. These results confirmed the formation of nucleosome units as compared with the control cell line, indicating the death of the cells via apoptosis [24]. No ladder was observed in the control cells. Thus, the cytotoxic effect of the treatment with the Pt complex allowed the crumbling of apoptotic DNA, and cell death was possibly mediated via an apoptotic mechanism. The replication of DNA molecule can be inhibited through a breakdown in its molecule, which is caused by inter-nucleosome cleavage accompanied with apoptosis, proposing that linalool-Gold nanoparticles GNP (LG) and linalool-GNP-CALNN peptide (LGC) can evoke fragmentation and damage in nucleic acid via different free radicals [25]. The reason behind this kind of DNA cleavage is the cleavage among the exposed linker regions of nucleosomes in response to the stimulation of endogenous endonucleases [26].

### 2.4. Effect of the Pt(II) Complex in Mitochondrial Membrane Potential of Caov-3 and HT-29 Cells

**Figure 4:**
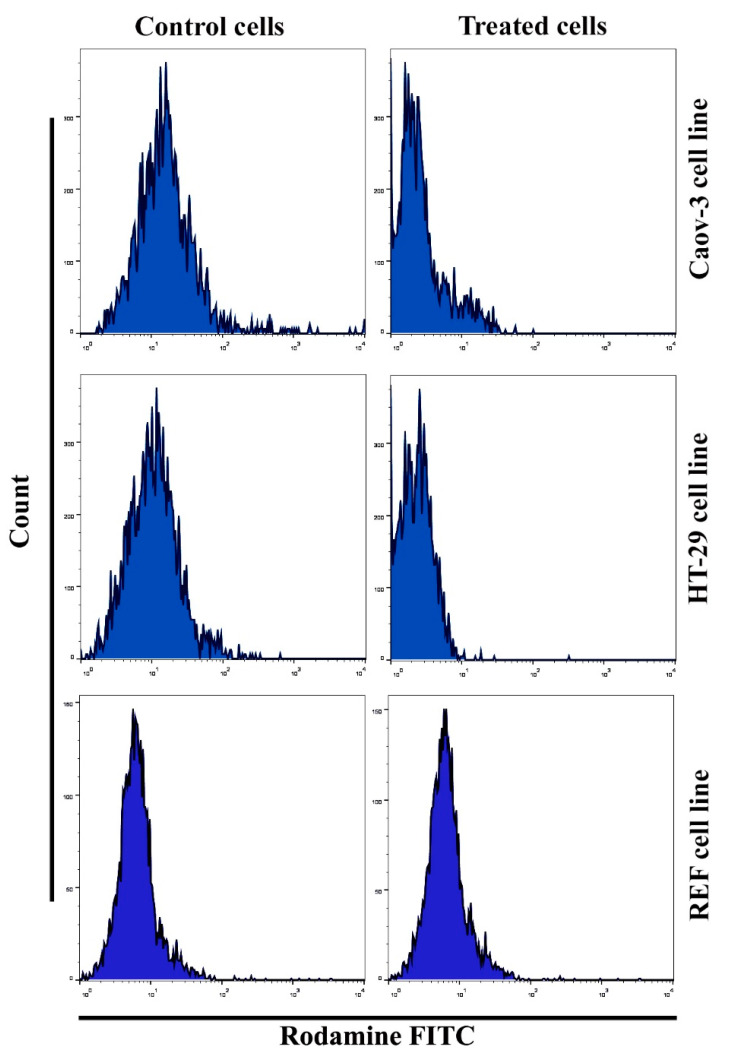
Effect of [Pt(H_2_L)(PPh_3_)] complex on the loss of mitochondrial membrane potential.

**Figure 5:**
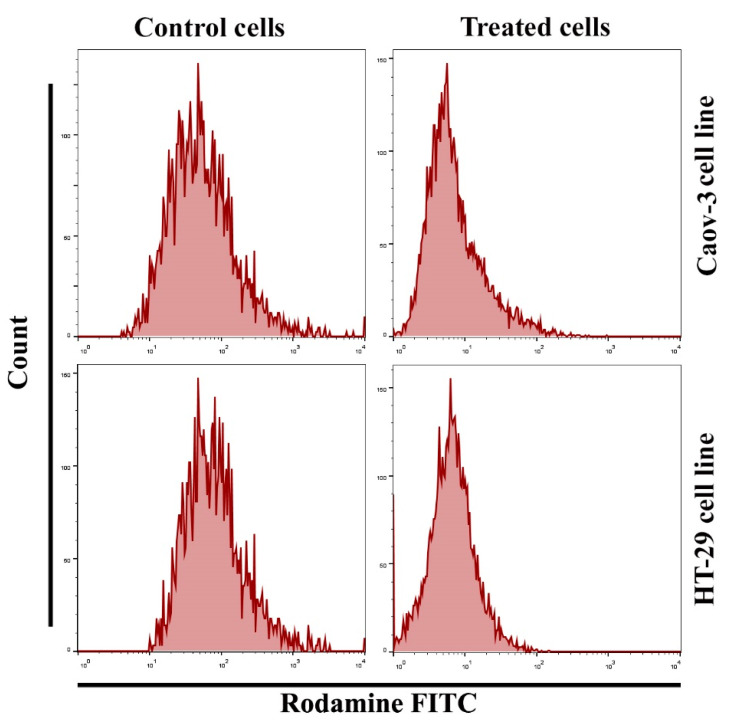
Effect of cisplatin on the loss of mitochondrial membrane potential.

The mitochondria play a crucial and essential role in inducing apoptotic activities through cell death stimulation. Changes in this organ are characterized by the loss of its membrane potential (∆ψm) and the release of cytochrome c protein into the cytoplasm, leading to caspase-3 via the caspase-9 pathway. In this study, apoptosis was detected using flow cytometry assay according to the manufacturer’s protocol. An important and relevant marker for the apoptotic cell death process is the reduction in mitochondrial membrane potential. The mitochondrial membrane potential level was examined after staining the cells with the Rh123 probe via flow cytometry. The number of apoptotic cancer cells after Pt(II) complex treatment was calculated. A significant increase in apoptosis due to Pt(II) complex treatment was observed in Caov3 and HT-29 cells (Figure 4), while no change observed after treated the normal cell line Rat embryonic fibroblast (REF) cells. The same results were seen after treated cancer cells with cisplatin as shown in (Figure 5). A significant decrease in cancer cells treated with the Pt(II) complex at IC_50_ for 24 h was noted in the Rh123 stain, which corresponded to the depletion of mitochondrial membrane potential compared with the untreated control cell classes.

### 2.5. Cell Cycle Analysis

**Figure 6:**
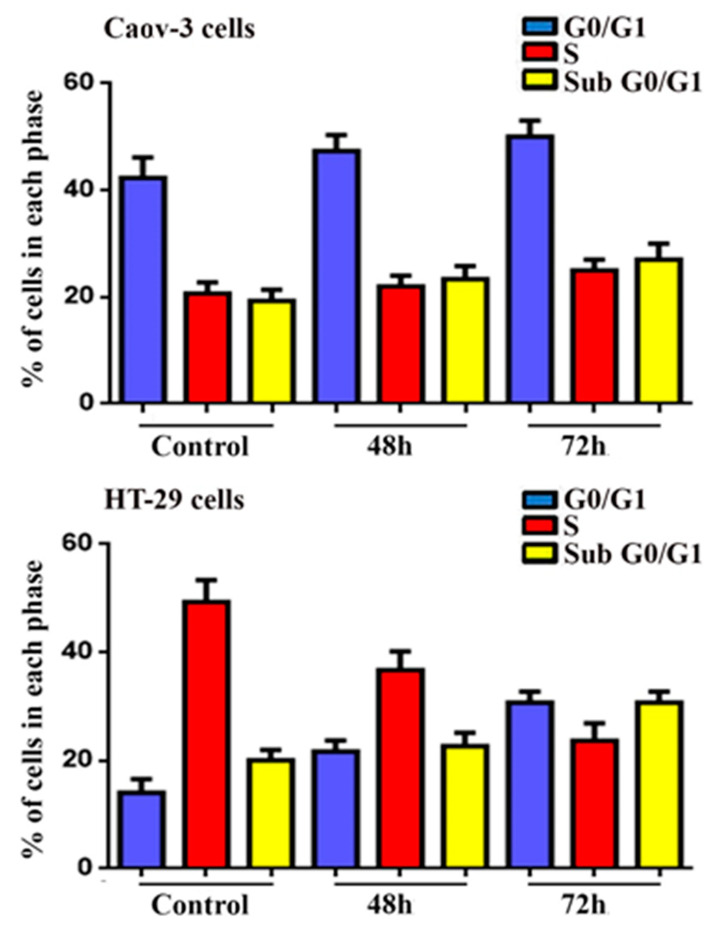
Cell cycle analysis of Caov-3 and HT-29 cell line after treatment with platinum(II) complex at different times as indicated. Bars show mean ± SEM.

As shown in Figure 6, the DNA content of cells was replicated during the synthesis phase (S phase), whereas the cells in the Gap0 (G0) and Gap1 (G1) phases had unreplicated nucleic acid. Meanwhile, the Gap2 (G2) and mitosis (M) phases showed replicated nucleic acid. In the flow cytometry analysis, the sub-G0/G1 peak was observed in histograms due to the degradation of nucleic acid and the formation of hypodiploid. Sub-G0/G1 is a selected marker of cellular apoptosis [27]. Analysis by flow cytometry of the ovarian adenocarcinoma and colorectal adenocarcinoma was carried out after respective IC_50_ treatments of the Pt complex for 48 and 72 h. The histogram showed a gradual increase in the sub-G0/G1 phase during the incubation period. The results showed the capacity of the Pt complex to induce the apoptosis pathway of Caov-3 and HT-29 cancer cells via the G0/G1 phase [28].

### 2.6. Pt(II) Complex Induces the P53 and Caspase-8 Pathways

**Figure 7:**
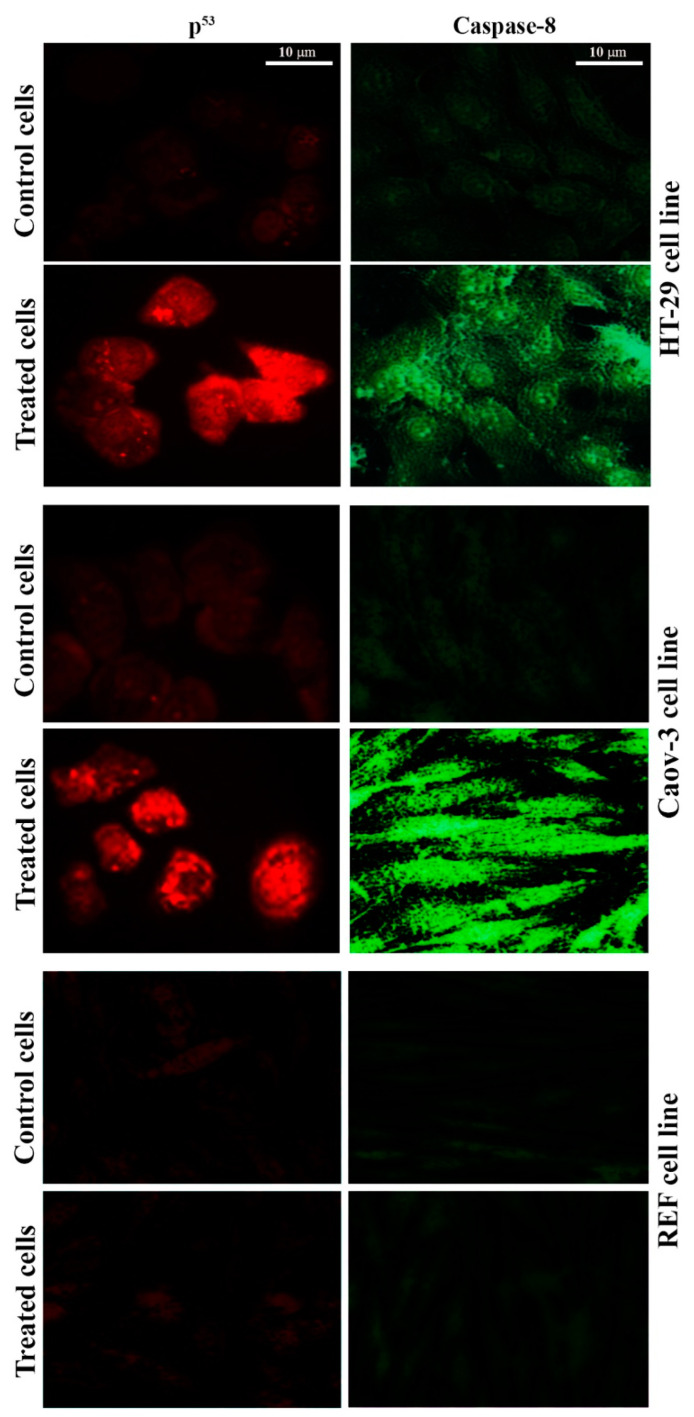
[Pt(H_2_L)(PPh_3_)] complex inducing apoptosis through p53 and Caspase-8 pathway. Immunofluorescence results of p53 and Caspase-8. Cells were untreated (control), or treated with [Pt(H_2_L)(PPh_3_)] complex as indicated. Cells were fixed, permeabilized, and then stained with primary and secondary antibodies. Scale bar 10 µm.

**Figure 8:**
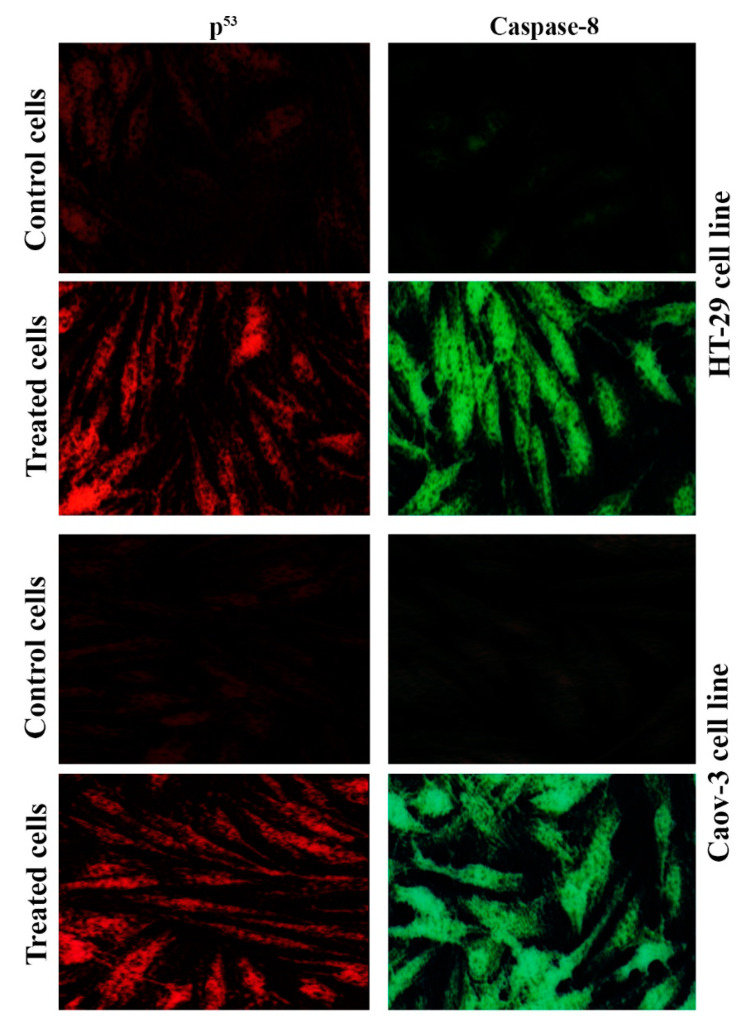
Cisplatin induces apoptosis through p53 and Caspase-8 pathway. Immunofluorescence results of p53 and Caspase-8. Cells were untreated (control), or treated with cisplatin as indicated. Cells were fixed, permeabilized, and then stained with primary and secondary antibodies. Scale bar 10 µm.

The tumor suppressor p53 gene and caspase enzymes are helpful in the regular monitoring of cells, and they prevent them from transforming into cancerous cells. When a cell shows any pattern of malignancy, an activation process of the mechanisms responsible for DNA repair is initiated for the restoration of the altered DNA [29,30]. To confirm that the Pt(II) complex caused the induction of p53 and caspase-8, an immunofluorescent test was performed. Figure 7 demonstrates p53 and caspase-8 induction in the cell lines following treatment with the Pt(II) complex. The fluorescence of p53 and caspase-8 was very low in the control cells, but p53 and caspase-8 immunofluorescent signaling were very clear in the treated Coav-3 and HT-29 cells. The signal was disappeared when the normal cell line REF was treated with the same complex. While the results demonstrated that cisplatin induces apoptosis in Caov-3 and HT-29 cell lines through the p53 and caspase-8 pathway as shown in Figure 8. These results suggested that the Pt(II) complex can induce apoptosis via p53 and caspase-8.

## 3. Materials and Methods

### 3.1. Preparation and Characterization of the Pt Complex

The Pt(II) complex preparation and characterization methods were identical to those previously described [17].

### 3.2. Maintenance of Cell Cultures

Caov-3 and HT-29 cancer cell lines were used. These cell lines were kept in RPMI-1640 in the presence of fetal bovine serum (10%), penicillin (100 units mL^−^^1^), and streptomycin (100 µg mL^−^^1^). Cells were passaged in trypsin-EDTA, reseeding was at 80% confluence (twice a week), and incubation was at 37 °C [31].

### 3.3. MTT Assay

The cytotoxicity test was carried following prior methods [32]. Cells were seeded at 1 × 10^5^ cells mL^−^^1^ in wells of microtiter plates supplied with RPMI and permitted to adhere overnight. Dilutions of the platinum complex (0.39–50 μg mL^−^^1^) were prepared. The tested compound’s solutions were added in triplicate and incubated for 24, 48 and 72 h. Thereafter, the MTT solution was added to the cells. The medium was aspirated following the incubation period, and the DMSO solution was added to the wells. Absorption was calculated on a microplate reader for each well under a wavelength of 492 nm. Regression analysis was conducted to extract the concentration for 50% inhibition of cell growth (IC_50_).

### 3.4. Double Staining with Acridine Orange (AO)–Propidium Iodide (PI)

The ability of the platinum complex to induce apoptosis was investigated using AO/PI. In brief, after 24 h of seeding cells on 12-well glass slides, they were exposed to the IC_50_ preparations of the tested compound for 24, 48, and 72 h. Dual fluorescent dyes were inserted into each well after washing twice with PBS. To observe the cells, a fluorescent microscope was used [33].

### 3.5. Nucleic Acid Fragmentation Induction

DNA fragmentation analysis was performed based on the method in the kit guide of magnesium tissue culture for DNA extraction. Cell lines were exposed to IC_50_ concentrations of the compounds for 48 and 72 h. Cells with a density of 1.5 × 10^6^ cell mL^−^^1^ were used for this experiment. After reaching the confluence, the cells were detached and suspended in PBS. Centrifugation (1200 rpm, 4 °C, and 10 min) was performed to remove the media, followed by dissolving with DNA loading buffer and enforcement to agarose gel. Electrophoresis was run following staining with ethidium bromide. Nucleic acid fragmentation was observed by using a UV illuminator device [34].

### 3.6. Cell Cycle Investigation via Flow Cytometry

Flow cytometry was used to investigate the cell cycle in exposed ovarian adenocarcinoma and colorectal adenocarcinoma (Caov-3 and HT-29) cell lines treated with the Pt complex. In brief, cells (5 × 10^4^ cells mL^−^^1^) were exposed to the IC_50_ concentration of the Pt complex for the three-time duration (24, 48, and 72 h). After cell fixation, washing with PBS was conducted to remove excess ethanol. Propidium iodide (PI; 10 mg mL^−^^1^) was used to stain the exposed cells for 1 h at 37 °C. RNase A (10 mg mL^−^^1^) was employed to prevent the PI stain from binding with DNA molecules. The DNA content of the treated cells was investigated by flow cytometry [35].

### 3.7. Potential Assay of the Mitochondrial Membrane

Rh123, a fluorescent dye repossessed by lively mitochondria without cytotoxicity, determines the critical apoptotic events in cells treated with the Pt complex. This dye was used before and after treatment with the Pt complex to study the membrane potential of mitochondrial cells. In brief, 24 h seeding of cells in 96-well plates was followed by treatment with the IC_50_ dose of the extracted active compound and staining with Rh123 dye at 2.5 M for 1 h at 37 °C. The cells were detached by 0.5% trypsin-EDTA and then centrifuged at 300 rpm for 5 min. Cells were suspended in FACS buffer and calculated by flow cytometry, and histograms were created [36].

### 3.8. Immunofluorescence Assay

In brief, cells were first processed by washing with PBS, fixation with PFA (4%, 30 min, RT), permeabilization with Triton-X (0.5%, 30 min, RT), and blocking with normal goat serum (10%, 30 min). Subsequently, cells were treated with 1 µg mL^−^^1^ of each of the primary antibodies of anti-p53 and anti-caspase-8 (24 h at 4 °C) and washed with PBS three times. Cells were treated with 1 mg mL^−^^1^ of each of the secondary antibodies (Alexa Fluor 488-conjugated goat anti-rabbit IgG or Alexa Fluor 568-conjugated goat anti-mouse IgG) for 2 h at RT. After additional washing of the cells three times using PBS, they were examined under a fluorescent microscope [37].

### 3.9. Statistical Analysis

Unpaired *t*-test (GraphPad Prism 6, San Diego, CA, USA) was employed to statistically process the data, which were shown as the mean ± SEM of the three replicates per experiment [38].

## 4. Conclusions

This work provides a model of a Pt complex with thiocarbohydrazone ligand playing a dianionic role in chelating platinum. The ligand provides two tridentate binding pockets that could be of the NNN or NNS sort based on the geometry of the ligand, due to the existence of two indolic rings in the framework. The platinum(II) complex had extraordinary cytotoxic effects on ovarian adenocarcinoma and colorectal adenocarcinoma and provided a confirmation to a role of intrinsic pathways in the induction of apoptosis in these cells. Additionally, further in vitro and in vivo experiments are needed to fully evaluate the potential of this compound in terms of the optimal dose, route of administration, and usefulness as an efficient chemotherapeutic drug in treating different types of cancers.

## Data Availability

The data presented in this study are available on request from the corresponding author.
